# Single nucleotide polymorphisms in *A4GALT* spur extra products of the human Gb3/CD77 synthase and underlie the P1PK blood group system

**DOI:** 10.1371/journal.pone.0196627

**Published:** 2018-04-30

**Authors:** Radoslaw Kaczmarek, Katarzyna Szymczak-Kulus, Anna Bereźnicka, Krzysztof Mikołajczyk, Maria Duk, Edyta Majorczyk, Anna Krop-Watorek, Elżbieta Klausa, Joanna Skowrońska, Bogumiła Michalewska, Ewa Brojer, Marcin Czerwinski

**Affiliations:** 1 Laboratory of Glycobiology, Hirszfeld Institute of Immunology and Experimental Therapy, Wroclaw, Poland; 2 Faculty of Physical Education and Physiotherapy, Opole University of Technology, Opole, Poland; 3 Department of Biotechnology and Molecular Biology, University of Opole, Opole, Poland; 4 Regional Centre of Transfusion Medicine and Blood Bank, Wroclaw, Poland; 5 Regional Centre of Transfusion Medicine and Blood Bank, Katowice, Poland; 6 Department of Immunohaematology and Immunology of Transfusion Medicine, Institute of Haematology and Blood Transfusion, Warsaw, Poland; University of Insubria, ITALY

## Abstract

Contrary to the mainstream blood group systems, P1PK continues to puzzle and generate controversies over its molecular background. The P1PK system comprises three glycosphingolipid antigens: P^k^, P1 and NOR, all synthesised by a glycosyltransferase called Gb3/CD77 synthase. The P^k^ antigen is present in most individuals, whereas P1 frequency is lesser and varies regionally, thus underlying two common phenotypes: P_1_, if the P1 antigen is present, and P_2_, when P1 is absent. Null and NOR phenotypes are extremely rare. To date, several single nucleotide polymorphisms (SNPs) have been proposed to predict the P_1_/P_2_ status, but it has not been clear how important they are in general and in relation to each other, nor has it been clear how synthesis of NOR affects the P_1_ phenotype. Here, we quantitatively analysed the phenotypes and *A4GALT* transcription in relation to the previously proposed SNPs in a sample of 109 individuals, and addressed potential P1 antigen level confounders, most notably the red cell membrane cholesterol content. While all the SNPs were associated with the P_1_/P_2_ blood type and rs5751348 was the most reliable, we found large differences in P1 level within groups defined by their genotype and substantial intercohort overlaps, which shows that the P1PK blood group system still eludes full understanding.

## Introduction

**Fig 1:**
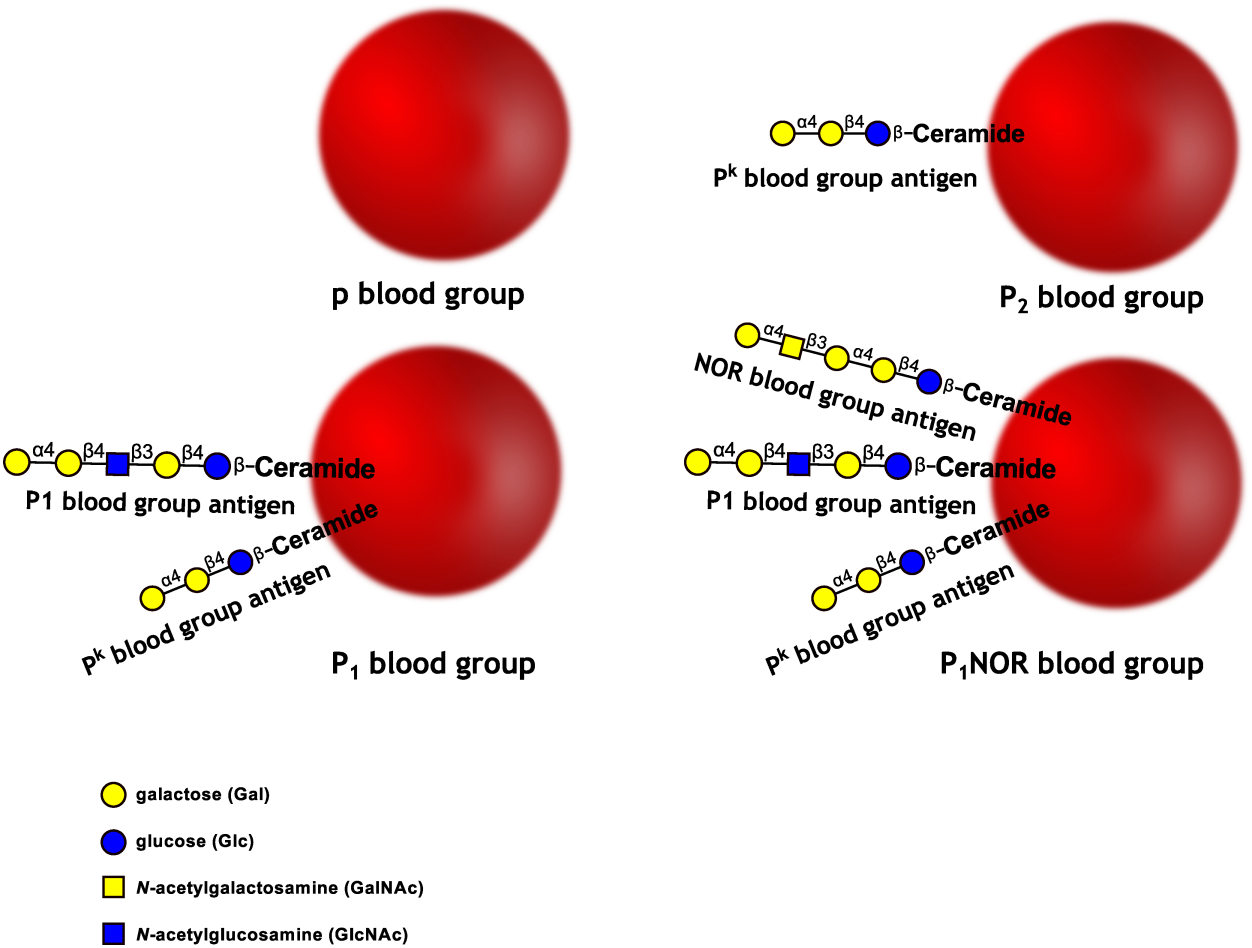
Schematic representation of the three glycosphingolipid antigens and phenotypes of the human P1PK blood group system.

Despite great strides made to understand the molecular background of human blood groups, the P1PK blood group system continues to puzzle. The difference between P_1_ and P_2_ (the two common P1PK phenotypes) red blood cells has been known since 1927, when Landsteiner and Levine found that rabbits immunized with human erythrocytes produced antibodies reacting with an antigen then named P and now called P1[1]. Since then, the P blood group system has been renamed P1PK (International Society of Blood Transfusion system 003), and while knowledge about the antigens belonging to that system has grown considerably, its molecular background is still far from being completely elucidated. The P1PK blood group system consists of three glycosphingolipid antigens: P^k^ (Gb3, CD77), P1 and NOR[2]. The P^k^ antigen is expressed on RBCs of most individuals (except in the null phenotype, denoted p), whereas P1 varies in different populations: from 30% in Japanese to 80% in Caucasians, to 94% in Blacks, thus underlying two common phenotypes: P_1_, if the P1 antigen is present, and P_2_, if P1 is absent[3]. The structures of the antigens belonging to the P1PK blood group system and phenotypes linked to these antigens are shown in Fig 1.

While it is well-established that the P^k^ antigen is synthesised by Gb3/CD77 synthase (α1,4-galactosyltransferase, P1/P^k^ synthase, encoded by *A4GALT*)[4], P1 has only recently been unequivocally shown to be a product of the same enzyme[5]. In addition, c.631C>G mutation in *A4GALT*, leading to p.Q211E substitution in Gb3/CD77 synthase (rs397514502), alters the enzyme specificity, rendering it able to synthesise all the three P1PK system antigens: P^k^, P1 and NOR[6]. This makes Gb3/CD77 synthase a unique case of a promiscuous glycosyltransferase, radically unmoored from the old ‘one enzyme-one linkage’ tenet[7].

Glycosphingolipids containing Galα1,4Gal unit are targeted by bacterial adhesins, toxins and viruses. These glycosphingolipids may localise in distinct membrane microdomains and act as genuine or decoy receptors. Most prominently, the P^k^ antigen is the receptor for Shiga toxins and its membrane localisation determines the toxins’ fate upon internalisation. P^k^ may be masked from the binding proteins and its recognition depends on the structure of its ceramide moiety and membrane microenvironment, including cholesterol content[8]. The P^k^ antigen was once believed to be a centroblast differentiation marker, which is why it is also known as CD77[9,10]. Unlike P^k^, the P1 antigen seems to be restricted to the erythroid lineage. Elsewhere than on RBCs, P1 was shown to be present only on ovarian cancer cells[11]. P1PK blood type may play a role in helminth infections, because these worms may display the Galα1,4Gal unit on their glycoconjugates for molecular mimicry[2,12,13].

**Fig 2:**
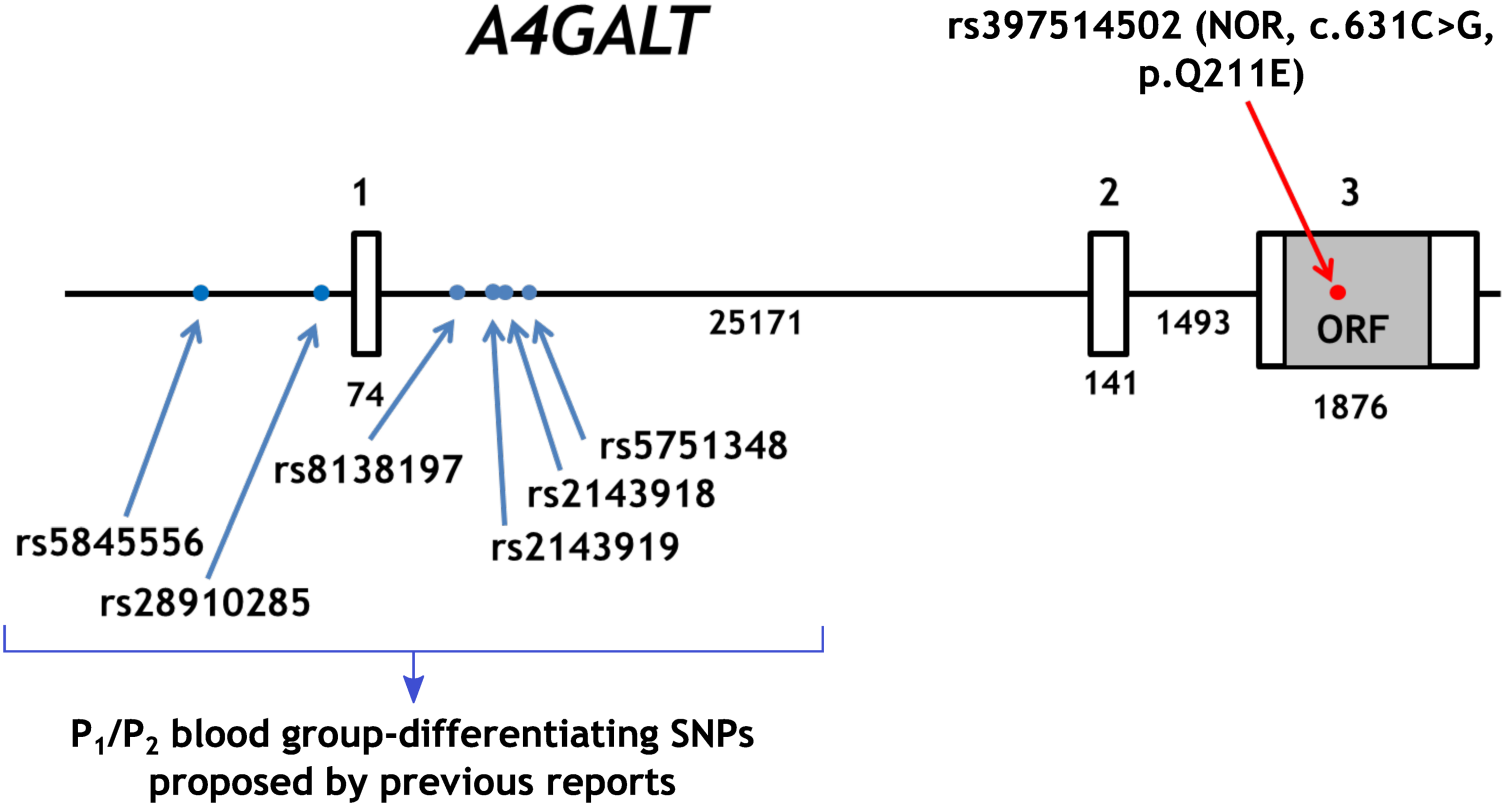
Structure of *A4GALT*, including the SNPs evaluated in this study.

The level of P1 antigen is related to the P_1_/P_2_ status and genotype at the *A4GALT* locus (*P*^*1*^*P*^*1*^, *P*^*1*^*P*^*2*^ or *P*^*2*^*P*^*2*^)[14,15]. The genetic background of theP_1_/P_2_ difference remained controversial for years, because it does not result from missense mutations and only one gene encoding Gb3/CD77 synthase exists in humans. Therefore, it is generally accepted that the P_1_/P_2_ status derives from varied *A4GALT* transcript levels. Several groups proposed different SNPs upstream from the coding region to underlie the P_1_/P_2_ difference. The SNPs rs5845556 (g.4501_4502insC, NG_007495.1) and rs28910285 (g.4892A/G, NG_007495.1) found by Iwamura et al (2003) were later found not to be correlated with the P_1_/P_2_ status. More recently, rs8138197[14] (g.7326C/T, NG_007495.1), rs2143918 (g.7837C/G, NG_007495.1), rs2143919 (g.7857T/G, NG_007495.1) and rs5751348[15] (g.8084G/T, NG_007495.1) found downstream of exon 1 of *A4GALT* were shown to be associated with the P_1_/P_2_ status (Fig 2). However, in either case, the statistical data presented in support of the identified SNPs were based on limited sample sizes, did not show the data distributions or effect size. Since differences in P1 antigen level may be confounded by a number of factors, such as *KLF1* expression level, extra scrutiny is desirable[16–19]. Also, none of the previous studies analysed the level of P1 antigen in NOR-positive RBCs, which warrants investigation, because the NOR antigen is synthesised by the same enzyme. To address the controversy over allelic variations of *A4GALT* gene expression and P_1_/P_2_ phenotypic differentiation[14,15], we analysed the effect of four SNPs (rs8138197, rs2143918, rs2143919, rs5751348) previously reported to determine the P_1_/P_2_ status on *A4GALT* transcript levels and cellular-scale quantity of the P1 and NOR antigens in a sample of 109 NOR-negative and NOR-positive Polish individuals. Importantly, we present the key results of this study in the form of univariate scatter plots, which faithfully represent distributions of continuous data, unlike still too often used bar plots[20].

## Materials and methods

### Sample preparation and P_1_/P_2_ blood typing

The study was approved by the Wrocław Medical University Bioethics Committee, Consent 641/2014, December 14, 2014. Blood (n = 109) from apparently healthy individuals was obtained following an informed consent according to the Declaration of Helsinki. Blood samples were collected on EDTA (for DNA extraction, flow cytometry and glycosphingolipid extraction) or on heparin (for RNA extraction), and washed RBCs were stored in CellStab low-ionic strength preservative solution (DiaMed, Cressier, Switzerland). DNA was extracted using Quick Blood DNA Purification Kit (EURx, Gdansk, Poland). RNA was prepared from the buffy coat using Human Blood RNA Purification Kit (EURx, Gdansk, Poland). The P_1_/P_2_ phenotype was determined by standard haemagglutination method using the human monoclonal anti-P1 antibody (S1 Table).

### Identification of the individual with PP1PK null (p) phenotype

During a routine antibody screening performed by the indirect antiglobulin test (IAT, DiaMed, Cressier, Switzerland) we found that the serum from a Polish blood donor agglutinated all panel RBCs (DiaMed, Cressier, Switzerland). To identify the antibody specificity, we employed an expanded panel of RBCs, including P—RBCs (the null phenotype of GLOB, a blood group system related to P1PK, 2 different samples) and p RBCs (the null phenotype of P1PK, 4 different samples) obtained from Serum Cells and Rare Fluid (SCARF) Exchange. These were used to test the donor’s plasma by the 2-stage enzyme (papain) test (DiaMed, Cressier, Switzerland), while the serum was tested by LISS IAT and 22°C saline tube tests. Very strong reactivity, agglutination or hemolysis, respectively, with all RBCs except P—and p phenotypes was observed. No reaction with the donor’s autologous RBCs was detected. The reactivity pattern indicated the anti-PP1Pk alloantibody specificity. The donor’s phenotype was confirmed by a monoclonal anti-P1 antibody and a panel of sera with different reactivities, including anti-P (1 sample) and anti-PP1Pk (3 different samples) from SCARF Exchange. Neither the monoclonal antibody nor any of the sera reacted with the donor’s RBCs, thus confirming his PP1PK null (p) phenotype.

### Analysis of the SNPs

Three DNA segments encompassing the studied SNPs were amplified using polymerase chain reaction (PCR). All primers are listed in S2 Table. PCR was performed using an MJ Mini gradient PCR apparatus (Bio-Rad, Hercules, CA, USA) in 20-μl reaction mixes containing 200 ng of genomic DNA, 0.2 mM dNTPs, Taq buffer with KCl (1:10 dilution), 1.5 mM MgCl_2_, 0.2 mM forward and reverse primers, and 1 unit of Taq polymerase (Fermentas, Vilnius, Lithuania). The PCR conditions are shown in S3 Table. The resulting DNA fragments were purified with a gel extraction kit (Gel-Out kit; A&A Biotechnology, Gdynia, Poland) and sequenced using the amplification primers. The genotypes (*P*^*1NOR*^*P*^*2*^, *P*^*1*^*P*^*2*^, *P*^*1NOR*^*P*^*1*^, *P*^*1*^*P*^*1*^, *and P*^*2*^*P*^*2*^) were assigned based on the SNP that best correlated with the P_1_/P_2_ blood typing and the c.631C/G status. Nucleotide differences between hetero- and homozygotes for individual P_1_/P_2_-related SNPs are shown in S1 Table. The *pp* genotype of the sole p individual in the cohort was confirmed previously [21].

### Quantitative analysis of transcripts

The complementary DNAs (cDNAs) were synthesised using 240 ng of RNA and SuperScript III First-Strand Synthesis kit (Life Technologies, Carlsbad, CA, USA) with oligo(dT) primers. Quantitative polymerase chain reaction (qPCR) was performed on 3 μl of cDNA using the 7500 Fast Real-Time PCR System (Life Technologies), according to the manufacturer’s instructions. A predesigned TaqMan assay targeting exon 2–3 boundary (Hs00213726_m1; Life Technologies) was used. Transcript quantities were normalised to ACTB (β-actin) endogenous control (assay Hs99999903_m1). All samples were run in triplicates. *P*^*2*^*P*^*2*^ samples were used as the calibrator. Data were analysed using Sequence Detection software Version 1.3.1 (Life Technologies). The real-time PCR conditions are shown in S4 Table.

### Antibodies

The human anti-P1, mouse anti-P1 and goat anti-mouse IgG conjugated with fluorescein isothiocyanate (FITC) antibodies were purchased from Immucor Inc. (Norcross, GA, USA), Ce-Immundiagnostika (Eschelbronn, Germany) and Santa Cruz Biotechnology (Dallas, TX, USA), respectively. The mouse monoclonal anti-NOR antibody, nor118 was obtained in our laboratory before and used as a diluted culture supernatant[22]. The goat anti-human IgM conjugated with FITC, biotinylated anti-mouse antibody and biotinylated anti-human antibody were purchased from Pierce (Rockford, IL, USA).

### Flow cytometry

0.5% RBC suspensions were incubated with 100 μl appropriately diluted primary antibodies (human anti-P1 1:400, mouse anti-P1 1:200, anti-NOR 1:20) for 40 min on ice. Then the cells were washed (all washes and dilutions were done with PBS) and incubated with 100 μl (diluted 1:100) FITC-labeled anti-mouse IgM antibody) for 40 min on ice in the dark. The cells were washed and suspended in 750 μl of cold PBS, and analysed by flow cytometry using FACSCalibur (BD Biosciences, Franklin Lakes, NJ, USA). The number of events analysed was 10,000/gated cell population. The results analysis was carried out using Flowing Software (Perttu Terho, University of Turku, Turku, Finland)[23].

### Quantitative flow cytometric analysis of the P1 antigen

The Quantum (Bio-Rad, Hercules, CA, USA) bead populations with defined quantities of FITC diluted in PBS were used to plot calibration curves (mean fluorescence intensity versus Molecules of Equivalent Soluble Fluorochrome units). The cells were then analysed by flow cytometry and the antibody binding capacities (ABCs, the number of antibody molecules bound per cell) were calculated by interpolation from the calibration curve as described in the manufacturer’s protocol and based on the fluorophore-to-protein molar ratios of the FITC-antibody conjugates. Negative control results (secondary antibody only) were subtracted from the sample results to obtain specific antibody binding capacities.

### Lipid panels

Fasting lipid profiles (mg% total cholesterol, HDL and LDL) were measured by Diagnostyka (Krakow, Poland). None of the individuals in the study were treated with statins.

### Extraction and purification of glycosphingolipids

The isolation and fractionation of total glycosphingolipids and the orcinol staining were performed as described previously[24,25]. Lipids were extracted with chloroform/methanol from freeze-dried RBC ghosts. The neutral glycosphingolipids were separated from the phospholipids and gangliosides, purified in peracetylated form, de-O-acetylated, and desalted. Glycosphingolipid samples were solubilised in chloroform/methanol (2:1, v/v), applied to HPTLC plates (Kieselgel 60, Merck, Darmstadt, Germany), and developed with chloroform/ methanol/water (55:45:9, v/v/v). The dried plates were immersed in 0.05% polyisobutylmethacrylate (Aldrich, Steinheim, Germany) in hexane for 1 min, dried, sprayed with TBS (0.05 M Tris buffer, 0.15 M NaCl (pH 7.4)), and blocked in 5% BSA. For antibody assays, the plates were sequentially overlaid with 1) primary antibody diluted in TBS/1% BSA (TBS-BSA) for 1–1.5 h; 2) biotinylated goat anti-mouse Ig antibody conjugated with alkaline phosphatase (Dako, Glostrup, Denmark), diluted 1:1000 with TBS-BSA; 3) ExtrAvidin-alkaline phosphatase conjugate (Sigma-Aldrich, St. Louis, MO, USA) diluted 1:5000 with TBS/ BSA/0.2% Tween20 for 1 h; and 4) the substrate solution (nitro blue tetrazolium/5-bromo-4-chloro-3-indolyl phosphate, Sigma-Aldrich). Other details were as described previously[25,26]. Each HPTLC experiment was repeated three times.

### Statistical analysis

The data distributions were tested for normality using Shapiro-Wilk test and inspected visually on Q-Q plots. The Box-Cox transformation was employed to reduce skewness and allow the use of parametric tests[27]. The means for transformed data were backtransformed to obtain weighted means in the original scale. Two-tailed two-sample independent t-test (or Welch’s test in the case of *P*^*1NOR*^*P*^*2*^ versus *P*^*1*^*P*^*2*^ and *P*^*1NOR*^*P*^*1*^ versus *P*^*1*^*P*^*1*^ human anti-P1 ABCs, and *P*^*1NOR*^*P*^*1*^ versus *P*^*1*^*P*^*1*^ and *P*^*1*^*P*^*2*^ versus *P*^*2*^*P*^*2*^ mouse anti-P1 ABCs, because of unequal variances) was employed for intercohort ABC mean comparisons. The backtransformed means were used to calculate mean differences and their 95% confidence intervals. Hedges’ g was used as the standardised measure of effect size to account for the small sample sizes. The 95% confidence intervals of standardised effect sizes were calculated based on noncentrality parameters of noncentral-t distributions. A Pearson’s correlation test was run to evaluate the relationships between total cholesterol, HDL and LDL levels and anti-P1 ABCs (we chose the *P*^*1*^*P*^*2*^ cohort to avail of the largest sample size). The correlation coefficients were tested for significance using a two-tailed t-test. A χ^2^ test with one degree of freedom and a type I error of 0.05 was run to check if the *P*^*1*^*P*^*1*^, *P*^*1*^*P*^*2*^ and *P*^*2*^*P*^*2*^ genotypes were in the Hardy-Weinberg equilibrium. All the statistical tests were performed with a type II error of 0.20 and type I error of 0.05 (except for multiple comparisons). The type I error was adjusted for multiple comparisons using the Dunn–Šidák correction to 0.0127 in the case of human and mouse anti-P1 ABCs, to 0.0253 in the case of *A4GALT* transcript levels, and to 0.0085 in the case of Pearson’s correlation test. All analyses were carried out using the Real Statistics Resource Pack software (Release 5.1, Copyright 2013–2017, Charles Zaiontz, www.real-statistics.com) in Microsoft Office Excel environment (Microsoft Corp, Redmond, WA).

## Results

### SNPs that best predict the P_1_/P_2_ blood type

To evaluate the role of single nucleotide polymorphisms in the P_1_/P_2_ blood group differentiation, we determined SNPs rs8138197 (g.7326C/T, NG_007495.1), rs2143918 (g.7837C/G, NG_007495.1), rs2143919 (g.7857T/G, NG_007495.1) and rs5751348 (g.8084G/T, NG_007495.1) in 109 Polish individuals. The results are shown in S1 Table. There were 84 P1-positive (P_1_, including 10 NOR-positive), 1 p (null), and 24 P1-negative (P_2_) individuals. The P_1_/P_2_ status of 100 individuals (91,7% of the total number) could be consistently predicted using all 4 evaluated SNPs. rs2143919 showed the weakest association with the phenotype, as shown before[15]. In two cases, we found that rs8138197 predicted *P*^*1*^*P*^*1*^ homozygosity, while the other 3 SNPs suggested *P*^*1*^*P*^*2*^. These two individuals were left out of the further analysis. One *P*^*2*^*P*^*2*^ (according to all four SNPs) individual was typed P_1_ in the agglutination test. Frequencies of the three genotypes (*P*^*1*^*P*^*1*^, *P*^*1*^*P*^*2*^ and *P*^*2*^*P*^*2*^) were in the Hardy-Weinberg equilibrium (p = 0.1823).

### Anti-P1 antibody binding capacities of RBCs with different genotypes

**Fig 3:**
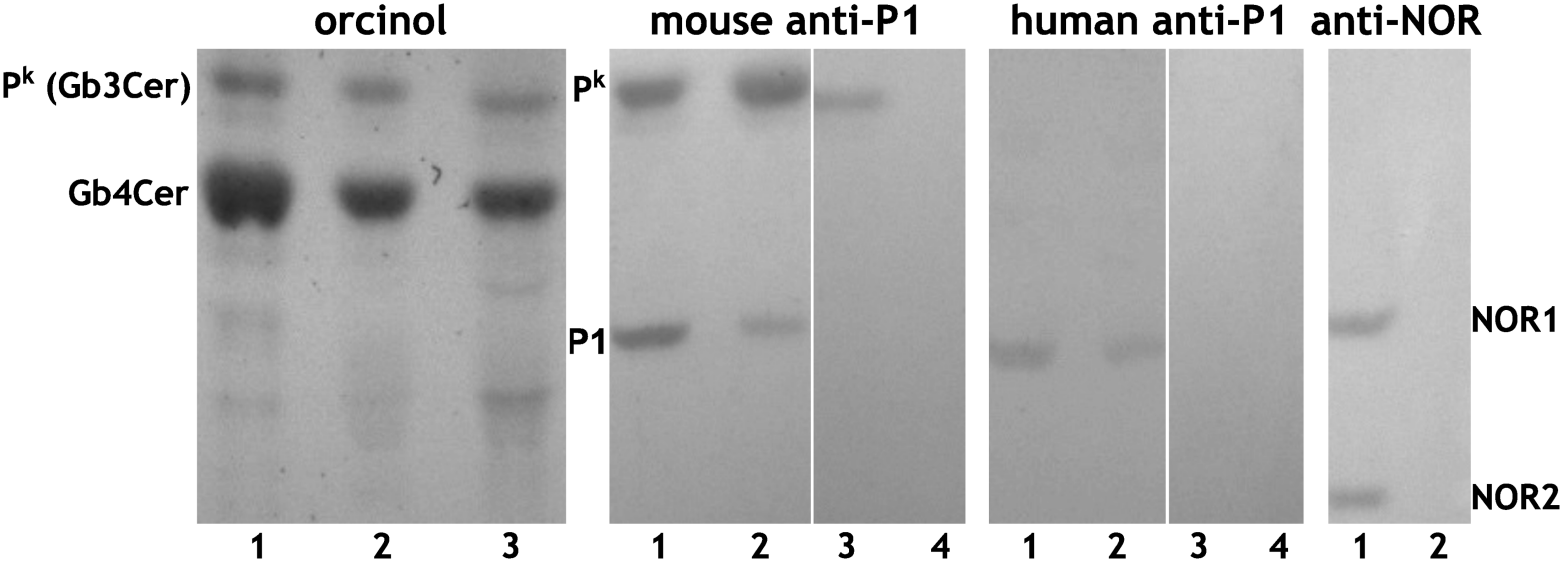
HPTLC analysis of total neutral glycosphingolipids extracted from red blood cells with different genotypes: *P*^*1NOR*^*P*^*1*^ (lane 1), *P*^*1*^*P*^*1*^ (lane 2), *P*^*2*^*P*^*2*^ (lane 3), and *pp* (lane 4). The image was uniformly treated with a gamma correction tool (IrfanView 4.38) to improve visibility. Individual panels represent separate silica plates, which were cropped and realigned for clarity. Full-length plates with original gamma settings are presented in S1 Fig.

Since we used two different (mouse and human) anti-P1 antibodies and one anti-NOR antibody, we performed an HPTLC analysis of neutral glycosphingolipids isolated from RBCs of different phenotypes (Fig 3). We found that the mouse monoclonal anti-P1 antibody (clone 650) binds to both P1 and P^k^ antigens, while the human anti-P1 monoclonal antibody (clone P3NIL100) binds to the P1 antigen only. The mouse monoclonal anti-NOR antibody (nor118) recognized only the NOR antigen represented by NOR1 and NOR2 glycosphingolipids. NOR1 and NOR2 contain the same terminal disaccharide, recognized by nor118, and emerge in the same pathway [28].

**Fig 4:**
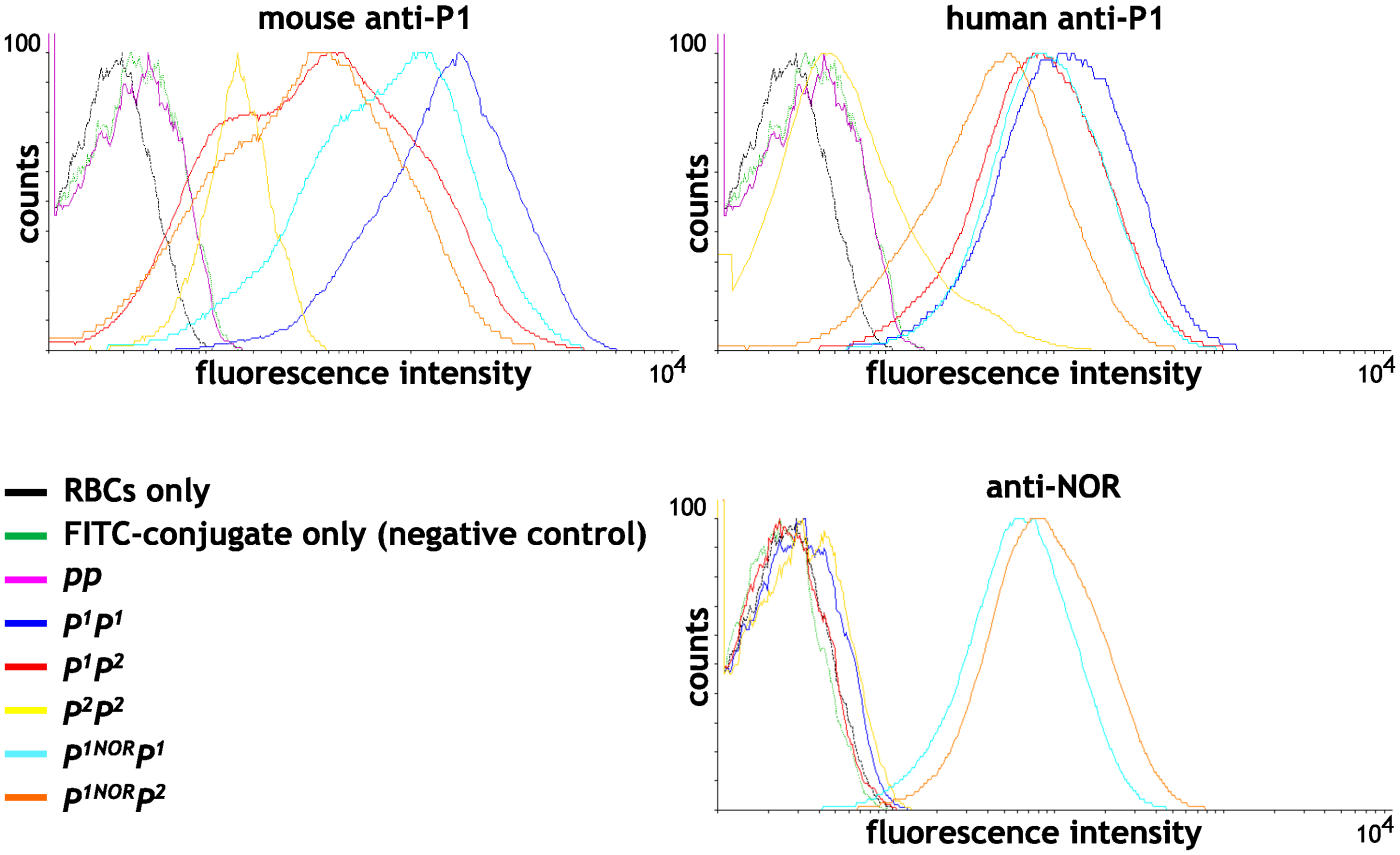
Flow cytometric analysis of the P1 and NOR antigens on RBCs with different genotypes. The fluorescence intensity is in log scale.

**Fig 5:**
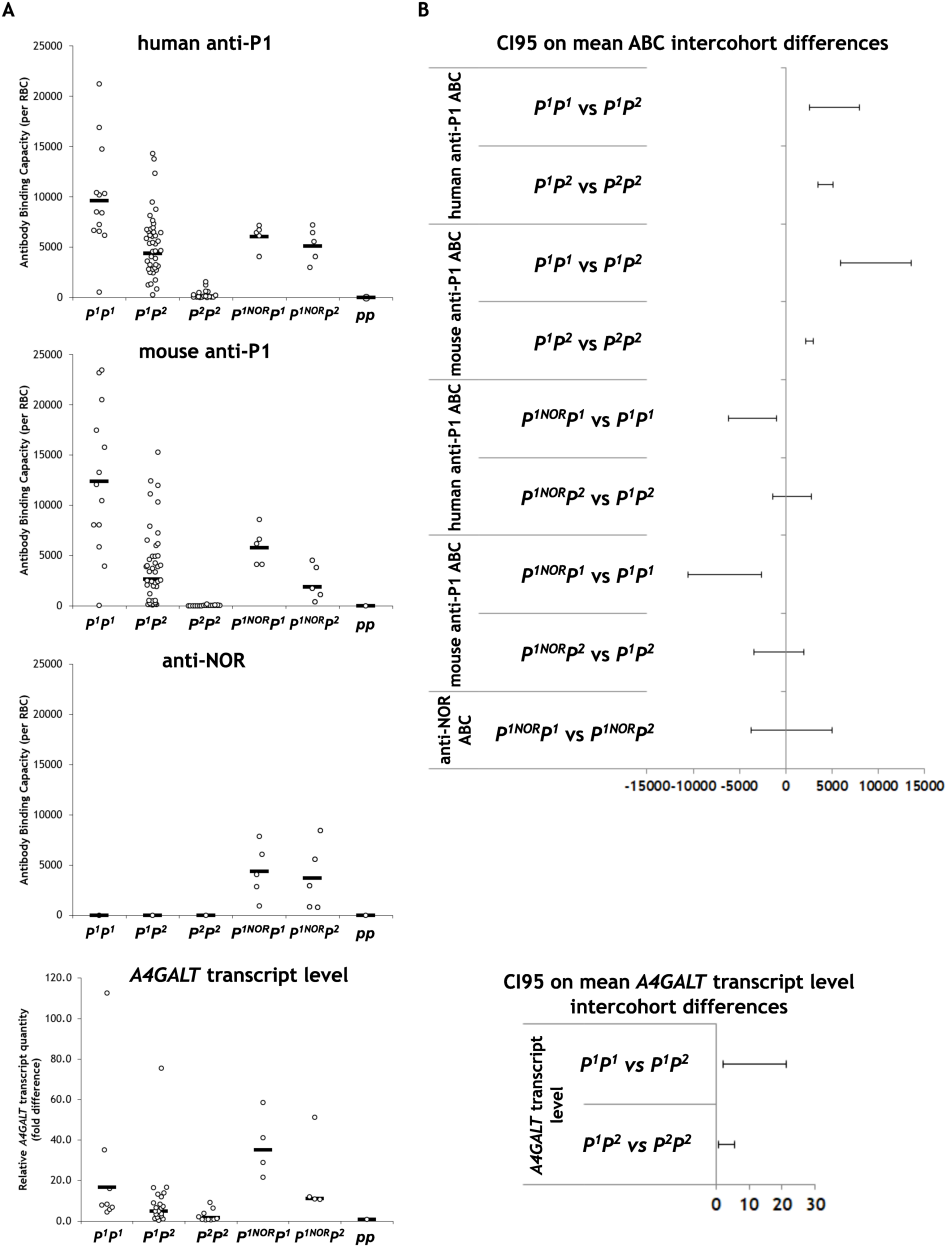
Scatter plots of RBC anti-P1 and anti-NOR antibody binding capacities (A) and relative *A4GALT* transcript levels in individuals with different genotypes and 95% confidence intervals on the intercohort mean differences (B).

In the quantitative flow cytometry (qFCM) analysis using the anti-P1 antibodies, *P*^*1*^*P*^*1*^ and *P*^*2*^*P*^*2*^ RBCs showed the highest and the lowest antibody binding capacities (ABC), respectively, while *pp* RBCs showed no binding at all (Fig 4). Scatter plots of ABC for all but the *P*^*2*^*P*^*2*^ group were skewed and widely distributed, revealing substantial intercohort overlaps. In the case of mouse anti-P1 antibody (clone 650), the weighted means (obtained by adjusting for skewness) of ABC were 12350, 2597, 11, 5735, 1850 and 0 antibody molecules per RBC for *P*^*1*^*P*^*1*^ (n = 13), *P*^*1*^*P*^*2*^ (n = 42), *P*^*2*^*P*^*2*^ (n = 18), *P*^*1NOR*^*P*^*1*^ (n = 5), *P*^*1NOR*^*P*^*2*^ (n = 5), and *pp* (null) (n = 1) genotypes, respectively; in the case of human anti-P1 antibody (clone P3NIL100), the weighted ABC means were 9608, 4389, 139, 6028, 5093 and 0, respectively (Fig 5A).

In contrast to the results of HPTLC analysis, the flow cytometry experiments suggested that the mouse anti-P1 antibody did not bind the P^k^ antigen. In fact, RBCs of all the cohorts except *P*^*1*^*P*^*1*^ showed higher human anti-P1 ABC than the mouse anti-P1 ABC. This would not have been the case if the mouse antibody bound both P1 and P^k^, because P^k^ is one of the major neutral glycosphingolipids of the RBC, unlike P1, which is a minor component of the RBC membrane, if at all present. Despite its abundance, P^k^ was shown to be hardly detectable for binding proteins when anchored in the cell membrane, in contrast to the lankier P1[29]. Henceforth, we assumed that in the RBC flow cytometry, both the human and mouse antibody recognize P1 only.

### Precision of phenotyping using the four SNPs

The ABC means were compared between cohorts to check whether the effect of the P_1_/P_2_-differentiating SNPs is significant across the three genotypes (*P*^*1*^*P*^*1*^ versus *P*^*1*^*P*^*2*^ and *P*^*1*^*P*^*2*^ versus *P*^*2*^*P*^*2*^; NOR-positive individuals were excluded from this analysis to eliminate the potential confounding effect of the altered enzyme activity). The two anti-P1 antibodies performed consistently insofar that in the case of both reagents the mean differences were statistically significant between *P*^*1*^*P*^*1*^ and *P*^*1*^*P*^*2*^, and *P*^*1*^*P*^*2*^ and *P*^*2*^*P*^*2*^, with large effect sizes (Table 1). Wide 95% confidence intervals reflected dispersion of the data (Fig 5B). The post-hoc statistical power analysis revealed that in all cases the tests were sufficiently powered, well above the 80% benchmark.

**Table 1:** Statistics on the effect of rs5751348 SNP on the P1 antigen level.

Statistic	Compared cohorts					
	Human anti-P1 ABC		Mouse anti-P1 ABC		*A4GALT* transcript level	
	*P*^*1*^*P*^*1*^ versus *P*^*1*^*P*^*2*^	*P*^*1*^*P*^*2*^ versus *P*^*2*^*P*^*2*^	*P*^*1*^*P*^*1*^ versus *P*^*1*^*P*^*2*^	*P*^*1*^*P*^*2*^ versus *P*^*2*^*P*^*2*^	*P*^*1*^*P*^*1*^ versus *P*^*1*^*P*^*2*^	*P*^*1*^*P*^*2*^ versus *P*^*2*^*P*^*2*^
Mean difference	5219	4250	9753	2586	11.9	3.2
p	0.0003	4.6 x 10^−15^	4.5 x 10^−6^	9.6 x 10^−18^	0.0175	0.0116
95% CI for the mean difference	2520 to 7918	3441 to 5059	5932 to 13574	2178 to 2993	2.3 to 21.5	0.7 to 5.7
Effect size (g)	1.3	3.0	1.7	-	1.2	1.0
95% CI for the effect size	0.6 to 2.0	2.2 to 3.8	1.0 to 2.4	-	0.2 to 2.1	0.2 to 1.8
Post-hoc power	85%	99.99997%	85%	99.99997%	54%	70.3%

The averaged ratio of transcript levels for *P*^*1*^*P*^*1*^, *P*^*1*^*P*^*2*^ and *P*^*2*^*P*^*2*^ genotypes was 10:3:1 (Fig 5). The mean differences between *P*^*1*^*P*^*2*^ and *P*^*1*^*P*^*1*^ or *P*^*2*^*P*^*2*^ were statistically significant and revealed a strong effect of the SNPs on *A4GALT* transcript level. The tests of these differences were underpowered, because qPCR was carried out on samples from fewer individuals (8 *P*^*1*^*P*^*1*^, 21 *P*^*1*^*P*^*2*^ and 10 *P*^*2*^*P*^*2*^) than qFCM, due to insufficient quality of some RNA extracts. However, the results of qFCM using both anti-P1 antibodies and qPCR were consistent across all the three cohorts.

Thus, rs5751348[15] showed the strongest association with anti-P1 antibody binding capacity, although outliers were prominent insofar that ABCs of RBCs with different genotypes overlapped extensively.

### Influence of the NOR mutation on the activity of Gb3/CD77 synthase

The c.631C>G missense mutation (rs397514502) was shown to broaden the specificity of Gb3/CD77 synthase, rendering it able to catalyse the synthesis of both Galα1,4Gal (the terminal disaccharide of P1 and P^k^ antigens) and Galα1,4GalNAc moieties (the terminal unit of NOR antigen), but its influence on enzyme activity *in vivo* remained unknown. P1, P^k^ and NOR antigens (represented by NOR1 and NOR2 glycosphingolipids) were all detected in glycosphingolipids isolated from NOR-positive RBCs using the anti-P1 and anti-NOR antibodies (Fig 3).

The anti-P1 ABCs of RBCs from NOR-positive individuals with *P*^*1*^*P*^*1*^ genotypes (*P*^*1NOR*^*P*^*1*^) was reduced by 54% in comparison with NOR-negative *P*^*1*^*P*^*1*^ individuals, when measured using the mouse antibody, and by 37%, when measured using the human one. However, comparison of NOR-positive (*P*^*1NOR*^*P*^*2*^) and NOR-negative *P*^*1*^*P*^*2*^ individuals (*P*^*1*^*P*^*2*^) generated conflicting results with the mouse antibody showing 29% lower ABC of *P*^*1NOR*^*P*^*2*^ RBCs, yet the human one showing 16% higher ABC of these RBCs (Fig 5). While the differences between *P*^*1NOR*^*P*^*1*^ and *P*^*1*^*P*^*1*^ cohorts were statistically significant, the differences between *P*^*1NOR*^*P*^*2*^ and *P*^*1*^*P*^*2*^ were not (Table 2).

**Table 2:** Statistics on the effect of c.631C>G (NOR) mutation on the P1 antigen level.

Statistic	Compared cohorts				
	Human anti-P1 ABC		Mouse anti-P1 ABC		Anti-NOR ABC
	*P*^*1NOR*^*P*^*2*^ versus *P*^*1*^*P*^*2*^	*P*^*1NOR*^*P*^*1*^ versus *P*^*1*^*P*^*1*^	*P*^*1NOR*^*P*^*2*^ versus *P*^*1*^*P*^*2*^	*P*^*1NOR*^*P*^*1*^ versus *P*^*1*^*P*^*1*^	*P*^*1NOR*^*P*^*1*^ versus *P*^*1NOR*^*P*^*2*^
Mean difference	704	-3581	-747	-6615	647
p	0.4632	0.0094	0.5811	0.0029	0.74
95% CI for the mean difference	-1375 to 2783	-6144 to -1017	-3452 to 1959	-10602 to -2627	-3749 to 5043
Effect size (g)	-	-	0.3	-	0.2
95% CI for the effect size	-	-	-0.7 to 1.3	-	-1.0 to 1.4
Post-hoc power	6.4%	42%	8.4%	42%	5.9%

The mean anti-NOR ABCs were 4354 and 3707 for *P*^*1NOR*^*P*^*1*^ and *P*^*1NOR*^*P*^*2*^, respectively. The mean difference was not statistically significant.

### Plasma cholesterol as a confounder of P1 expression

**Fig 6:**
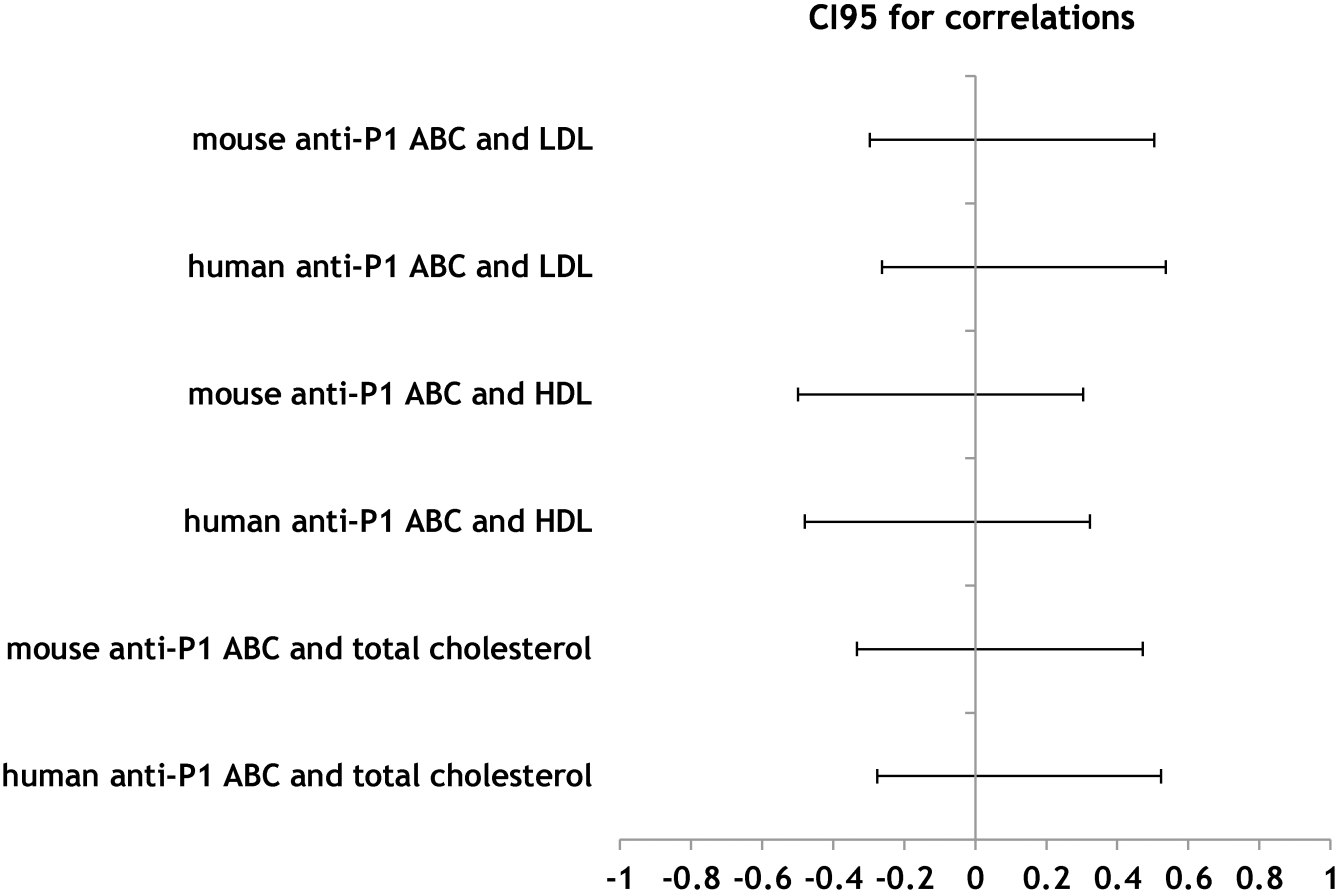
95% confidence intervals on Pearson’s correlation between RBC anti-P1 antibody binding capacities, HDL, LDL and total cholesterol.

Since it was postulated that the P1PK antigens localise primarily in lipid rafts, which are enriched in cholesterol, we checked total cholesterol, HDL and LDL plasma concentrations in individuals with known P1PK status. The data are shown in S1 Table. Although we did not find statistically significant correlations between plasma HDL or LDL levels and anti-P1 ABC, we found interesting if counterintuitive trends (Table 3, S2 Fig); the LDL- and HDL-ABC relationships were direct and inverse, respectively, and this pattern was consistent between tests involving the human and mouse anti-P1 ABC (Fig 6 and S2 Fig). The relationship between total cholesterol and anti-P1 ABC was positive. It is important to know that the correlation tests lacked statistical power for the observed effect sizes (the largest observed Pearson’s coefficient was 0.137 for the human anti-P1 ABC and LDL level). To confidently reject (or accept) the null hypothesis (no correlation) for the observed effect sizes, the sample would have to comprise over 410 individuals.

**Table 3:** Statistics on relationships between RBC anti-P1 antibody binding capacities, HDL, LDL and total cholesterol.

Statistic	Relationship					
	Human anti-P1 ABC and total cholesterol	Mouse anti-P1 ABC and total cholesterol	Human anti-P1 ABC and LDL	Mouse anti-P1 ABC and LDL	Human anti-P1 ABC and HDL	Mouse anti-P1 ABC and HDL
r	0.123	0.069	0.137	0.103	-0.079	-0.098
p	0.531	0.727	0.485	0.600	0.690	0.621
95% CI for r	-0.28 to 0.52	-0.33 to 0.47	-0.26 to 0.54	-0.30 to 0.50	-0.48 to 0.32	-0.50 to 0.30
Post-hoc power	9.5%	6.3%	10.6%	8.1%	6.8%	7.8%

## Discussion

Despite perennial efforts, P1PK remains the most elusive human blood group system. The manifold attempts to obtain a clear picture of how P1PK blood group differentiation ensues have been marred by the unorthodox activity of Gb3/CD77 synthase, the unusual ways in which genetic changes alter this enzyme’s activity, and the intertwined synthesis of the P1PK and other glycosphingolipid blood group antigens. While there is a consensus that *A4GALT* transcript level in P_1_ RBCs is higher than in P_2_, presumably because it leads to upregulation of Gb3/CD77 synthase (although it was not directly proven), the molecular background of that phenomenon remains obscure. There have been three reports showing that single nucleotide polymorphisms in the non-coding region of *A4GALT* may play a role in regulating transcription: Iwamura et al (2003), Thuresson et al (2011) and Lai et al (2014)[30,14,15]. Today, it is generally accepted that SNPs described by Iwamura et al play no role in P_1_/P_2_ polymorphism, while the SNPs proposed in the two other reports do matter, but so far it has not been completely clear how important they are in general and in relation to each other, because of statistical limitations of those studies. Importantly, the SNPs in question densely intersperse within a relatively short stretch of the gene, which adds to the challenge of studying their effects, because of a potential genetic linkage.

In this study, we set out to scrutinize the postulated effects of previously reported P_1_/P_2_-differentiating SNPs and map the influence of NOR mutation (c.631C>G in *A4GALT*) onto the P1 antigen level. Our results provide the strongest evidence yet that the four SNPs previously reported by Thuresson et al. and Lai et al. are related to the P_1_ or P_2_ phenotype[14,15], of which rs5751348[15] proved to be the most reliable, while the three other SNPs underperformed in terms of their predictive value. However, in contrast to the previous studies, the scatter plots of antibody binding capacities and transcript levels revealed wide and largely overlapping distributions. This shows that the SNPs in question, albeit important, cannot universally predict the phenotype and that the P1 antigen level may be confounded by other factors. One such potential confounder is the transcription factor EKLF (Erythroid Krüppel-Like Factor, encoded by *KLF1*), not least because rs5751348 is located in an EKLF consensus binding site[16]. The most recent studies implicated the transcription factors EGR1[31] and RUNX1[32] in regulation of *A4GALT* expression, and so P1 synthesis. The P1 antigen level may also be influenced by competition between Gb3/CD77 synthase and other glycosyltransferases for the same substrate, paragloboside, which is the direct precursor of P1, as well as by activities of enzymes acting upstream in its biosynthetic pathway[12]. Here, we looked at another, rarely addressed, potential confounder: the RBC membrane cholesterol content. It was shown that excess plasma membrane cholesterol may cloak glycosphingolipids, including the P^k^ antigen, from the binding proteins[8,29,33]. Most of these studies focused only on the P^k^ antigen’s role as a Shiga toxin receptor, which is perhaps why the cholesterol aspect has not crossed over to immunohaematologic studies on the P1PK system so far. However, P1 is likely to be subject to the same masking mechanisms as P^k^, although the longer sugar chain may limit the extent to which it can become stealth. To evaluate cholesterol as a potential confounder of the P1 antigen level, we looked at plasma total cholesterol, HDL and LDL concentrations. The parameters we chose to look at seemed to be good surrogates of the RBC membrane cholesterol content, because RBCs engage in the systemic cholesterol flux and RBC membranes exchange cholesterol with plasma lipoproteins [34–36]. This study lacked statistical power to assert or rule out the small effect of cholesterol level, yet it was interesting to see the tendency of LDL and HDL to be related positively and negatively with anti-P1 antibody binding capacities, respectively. Since HDL transports cholesterol from peripheral tissues to the liver, the reverse being the job of LDL, the inverse relationship of HDL with anti-P1 ABC and the direct relationship of LDL therewith is counterintuitive. However, this pattern was consistent across all comparisons and made more plausible by the direct relationship between total cholesterol and anti-P1 ABC. These surprising trends may suggest that the unique features of the RBC membrane, such as its unusual curvature, alter the way in which cholesterol affects crypticity of RBC antigens or that the P1 antigen membrane topology is different than expected and merit further study.

We also attempted to evaluate the effect of NOR mutation (c.631C>G) on activity of the Gb3/CD77 synthase *in vivo* and how it relates to our earlier studies *ex vivo* and *in vitro*[5–7]. In our study on a transfected cell line[7], activity of the consensus Gb3/CD77 synthase was higher than activity of the p.Q211E mutein, in contrast to our results with the purified recombinant variants of the enzyme. The results of this study suggest that the p.Q211E enzyme’s superpromiscuity comes at the cost of P1 synthesis. The use of quantitative flow cytometry assay allowed us to compare binding levels of anti-P1 and anti-NOR antibodies, regardless of differences in specificity and quantities of the fluorophore conjugated to the secondary antibodies. On that basis, it may be estimated that at the cost of one P1 molecule the p.Q211E mutein produces one NOR molecule. The disparate results of *in vivo* and *in vitro* studies are not entirely unexpected, since glycosyltransferases *in vivo* may enter homo- and heterooligomeric complexes and show altered enzyme activities[37–42]. It was demonstrated that Gb3/CD77 synthase may form such complexes as well, but in this case the functional relevance remains unknown[43,44]. Since all the statistical tests involving NOR-positive cohorts lacked power to pinpoint the effect sizes, these tests were subject to either type II or type M error. This problem could not be worked around by increasing the sample sizes, because of extreme rarity of the NOR-positive phenotype.

Notably, the sole *pp* (null) individual in the study revealed very low *A4GALT* transcript level despite his *P*^*1*^*P*^*1*^ homozygosity (for all the four SNPs). It was markedly lower than the minimal one in the *P*^*1*^*P*^*1*^ cohort and lower than the mean in the *P*^*2*^*P*^*2*^ cohort. The genetic background of that individual’s null phenotype was previously described[21]. It derives from a nonsense mutation in one allele and a missense mutation in the other, so the low transcript level may be caused by nonsense-mediated mRNA decay, which is a quality-control mechanism removing mRNAs with premature termination codons[45].

In summary, our results show that the SNPs rs8138197 and rs5751348 proposed by Thuresson et al and Lai et al[14,15], respectively, are strongly associated with the P_1_/P_2_ status, but rs5751348[15] offers the best predictive value, while rs8138197 underperforms. The growing importance of genetic testing quickly caught up with transfusion medicine and prompted the search for genetic markers that would replace standard haemagglutination assays, at least in complex scenarios, such as foetal testing[46]. In the case of P1PK blood group system, P_1_/P_2_ blood typing based on the known SNPs requires caution, because it lacks perfect accuracy. In addition, individuals with different genotypes show largely varying levels of the P1 antigen, which suggests that it may be strongly confounded by other (not necessarily genetic) factors. We also show for the first time how synthesis of all the three P1PK system antigens (P^k^, P1 and NOR) may alter the P_1_ phenotype, which affords a better understanding of the system and how the responsible enzyme, Gb3/CD77 synthase, operates *in vivo*. The promiscuous activity of this enzyme and its impact on the phenotype stand in stark contrast to how carbohydrate-active enzymes used to be viewed. While the complete picture of the P1PK blood group system seems to be closer than ever, uncertainty remains around indirect factors contributing to the phenotype, so the final chapter in the P1PK story is yet to be written.

## Supporting information

S1 Fig HPTLC analysis of neutral glycosphingolipids extracted from RBCs with different genotypes: *P*^*1NOR*^*P*^*1*^ (lane 1), *P*^*1*^*P*^*1*^ (lane 2), *P*^*2*^*P*^*2*^ (lane 3), and *pp* (lane 4). The image represents chemical staining and immunoverlays of silica plates from different chromatography runs before cropping and realignment.
(PDF)

S2 Fig Scatter plots of relationships between Box-Cox transformed RBC anti-P1 binding capacities, HDL, LDL and total cholesterol. (PDF)

S1 Table Phenotypes, genotypes, RBC antibody binding capacities and lipid profiles of recruited individuals. The colour-coding is explained beneath the table.
(PDF)

S2 Table Sequences of primers used for genotyping. (PDF)

S3 Table PCR conditions used for amplification of *A4GALT* fragments encompassing the studied SNPs. (PDF)

S4 Table Real-time PCR conditions used for *A4GALT* gene expression assays. (PDF)
