# Supplementary material for: Single nucleotide polymorphisms in A4GALT spur extra products of the human Gb3/CD77 synthase and underlie the P1PK blood group system
Source: PLoS One. 2018 Apr 30;13(4):e0196627. doi: 10.1371/journal.pone.0196627 (PMC5927444; doi:10.1371/journal.pone.0196627)

Supplementary Figure 1. HPTLC analysis of neutral glycosphingolipids extracted from RBCs of $P^{1NOR}P^{1}$ (lane 1), $P^{1}P^{1}$ (lane 2), $P^{2}P^{2}$ (lane 3), and *pp* (lane 4) genotypes. The image represents chemical staining and immunoverlays of silica plates from different chromatography runs before cropping and realignment.

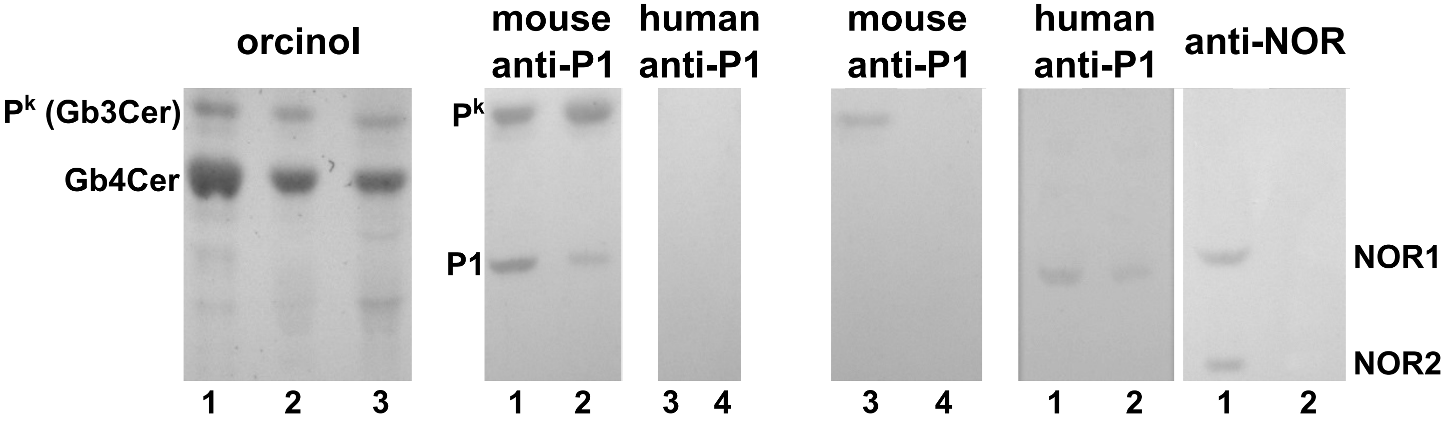

Supplement: S1 Fig — The image represents chemical staining and immunoverlays of silica plates from different chromatography runs before cropping and realignment. (PDF) [file pone.0196627.s001.pdf]
