# Supplementary material for: Single nucleotide polymorphisms in A4GALT spur extra products of the human Gb3/CD77 synthase and underlie the P1PK blood group system
Source: PLoS One. 2018 Apr 30;13(4):e0196627. doi: 10.1371/journal.pone.0196627 (PMC5927444; doi:10.1371/journal.pone.0196627)

Supplementary Figure 2. Scatter plots of relationships between Box-Cox transformed RBC anti-P1 binding capacities, HDL, LDL and total cholesterol.

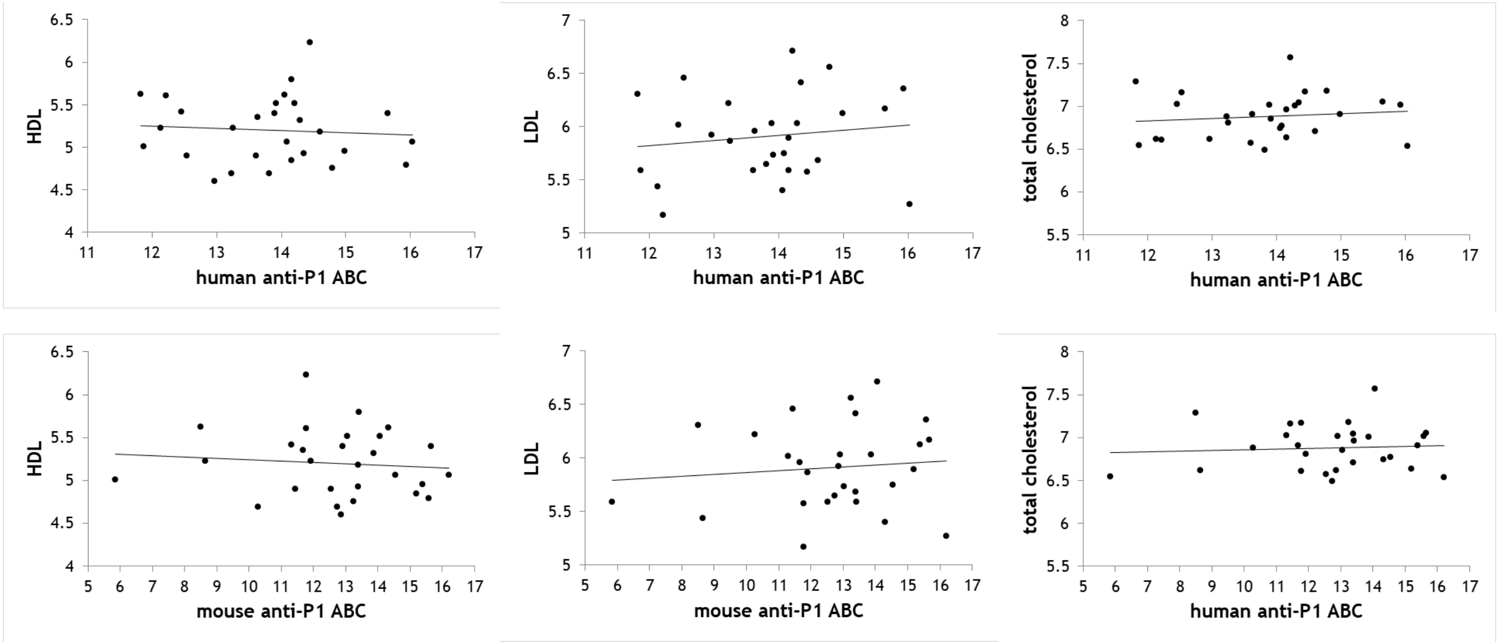

Supplement: S2 Fig — (PDF) [file pone.0196627.s002.pdf]
