# Supplementary material for: Single nucleotide polymorphisms in A4GALT spur extra products of the human Gb3/CD77 synthase and underlie the P1PK blood group system
Source: PLoS One. 2018 Apr 30;13(4):e0196627. doi: 10.1371/journal.pone.0196627 (PMC5927444; doi:10.1371/journal.pone.0196627)
Supplement: S1 Table — The colour-coding is explained beneath the table. (PDF) [file pone.0196627.s003.pdf]

Table S1. Phenotypes, genotypes, RBC antibody binding capacities and lipid profiles of recruited individuals. The colour-coding is explained beneath the table.

Sample	Agglutination				ABC			SNPs correlating with P_1/P_2 status				Lipid profile (mg%)		
	A	B	D	P1	h a-P1	m a-P1	a-NOR	rs8138197	rs2143919	rs2143918	rs5751348	Total chol	HDL	LDL
15	+	-	+	+	10416	23459		cc	cc	tt	gg	249	89	145
19	+	+	+	+	8387	20511		cc	cg	tt	gg			
29	-	-	+	+	14721	17441	0	cc	cg	tt	gg	220	69	132
39	-	-	+	+	6657	13273		cc	cc	tt	gg			
41	+	-	+	+	8473	15777		cc	cc	tt	gg			
45	-	-	+	+	16880	8038		cc	cg	tt	gg	169	57	89
47	-	+	+	+	7255	8011		cc	cc	tt	gg			
52	+	+	+	+	6561	10423		cc	cc	tt	gg	205	44	133
55	+	+	+	+	6168	5847		cc	cc	tt	gg	234	44	134
56	+	-	-	+	10331	23208		cc	cc	tt	gg	264	54	190
75					21226	12055		cc	cc	tt	gg			
84								cc	cc	tt	gg			
89	+	-	+	+				cc	cc	tt	gg			
90	-	+	+	+	10186	3913		cc	cc	tt	gg			
101	+	-	-	+	316	0		cc	cc	tt	gg			
110								cc	cc	tt	gg			
111					0	0	0	cc	cc	tt	gg			
1	+	-	+	+	9485	11106		ct	cg	tg	gt	191	56	119
4	+	-	+	+	8759	4598		ct	cg	tg	gt	224	49	155
6	-	-	+	+	6761	4936		ct	gg	tg	gt	197	97	85
10	+	-	+	+	6927	6513		ct	cg	tg	gt	280	81	170
11	+	-	+	+	6129	4214		ct	cg	tg	gt	185	81	93
16	+	-	+	+	6769	10299		ct	gg	tg	gt	162	52	103
17	-	+	-	+				ct	cg	tg	gt	188	43	98
18	-	-	+	+	13748	11979		ct	cg	tg	gt	204	50	137
20	+	+	+	+	3896	3874		ct	cg	tg	gt			
21	+	-	+	+	2448	471		ct	cg	tg	gt	238	87	133
22	+	-	+	+	3626	2505		ct	cg	tg	gt			
23	-	-	+	+	4083	3888		ct	cg	tg	gt	161	44	105
24	-	-	+	+	5572	3327		ct	cg	tg	gt			
25	+	-	+	+	2815	508		ct	gg	tg	gt	161	67	77
26	+	-	+	+	7143	5991		ct	cg	tg	gt	203	71	112
27	+	+	+	+	6071	3976		ct	cg	tg	gt	204	75	112
28	+	-	+	+	5430	2277		ct	cg	tg	gt	191	73	107
30	+	-	+	+	4572	1170		ct	cg	tg	gt	188	47	126
31	-	+	+	+	4630	2548		ct	cg	tg	gt	180	67	101
35	+	+	+	+				ct	cg	tg	gt	165	71	75
36	+	-	+	+	3376	2045		ct	gg	tg	gt	222	54	146
42	+	-	+	+	5376	3378		ct	cg	tg	gt	156	54	85
43	+	-	+	+	4600	6159		ct	cg	tg	gt			
44	-	-	+	+	14296	15286		ct	cg	tg	gt	153	60	69
46	+	-	+	+	3256	1927		ct	gg	tg	gt	205	76	111
48	+	-	+	+	8133	4887		ct	cg	tg	gt	170	65	90
49	+	-	+	+	5295	1895		ct	cg	tg	gt			
50	+	+	+	+	5868	3691		ct	cg	tg	gt	149	47	88
51	+	-	+	+	6565	7916		ct	gg	tg	gt	176	60	94
54	+	+	+	+	6498	7225		ct	cg	tg	gt	173	86	75
57	-	+	+	+	7317	4911		ct	cg	tg	gt	207	55	142
58	+	-	+	+	12338	12389		ct	cg	tg	gt	208	75	122
59	+	-	+	+	6424	3994		ct	cg	tg	gt			
60	+	-	+	+	2932	2396		ct	cg	tg	gt	160	85,8	65
72	-	-	+	+	2507	99		ct	cg	tg	gt	154	58	85
73	-	+	-	+	1761	93		ct	cg	tg	gt			
74	+	-	+	+	3103	528		ct	cg	tg	gt			
80					7619	2403		ct	cg	tg	gt	223	127	84
81							0	ct	gg	tg	tg			
85								ct	cg	tg	gt			
86								ct	cg	tg	gt			
87	-	+	+	+				ct	cg	tg	gt			
88	-	-	+	+				ct	cg	tg	gt			
92								ct	cg	tg	gt			
95	+	-	+	+	225	156		ct	cg	tg	gt			
96	-	-	+	+	1042	228		ct	cg	tg	gt			
97	-	-	+	+	554	108		ct	cg	tg	gt			
98	-	-	+	+	581	170		ct	cg	tg	gt			
99	+	+	-	+	410	84		ct	cg	tg	gt			
100	-	-	+	+	1067	286		ct	cg	tg	gt			
108								ct	cg	tg	gt			
109								ct	cg	tg	gt			
2	-	-	+	-	239	25		tt	gg	gg	tt	343	47	275
3	+	-	+	-	0	3		tt	gg	gg	tt	211	54	147
5	-	-	+	-	585	85		tt	gg	gg	tt	221	92	114
7	-	-	-	-	120	0		tt	gg	gg	tt	220	90	114
8	-	-	+	-	97	5		tt	gg	gg	tt			
12	+	+	+	-	46	1		tt	gg	gg	tt	200	56	136
32	+	+	-	-	41	1		tt	gg	gg	tt			
33	-	-	+	-	36	0		tt	gg	gg	tt			
34	+	-	+	-	14	0	0	tt	gg	gg	tt	172	61	97
38	+	-	+	+	1236	77		tt	gg	gg	tt	171	69	91
40	+	-	+	-	81	0		tt	gg	gg	tt			
53	-	-	+	-	468	14		tt	gg	gg	tt			
76					267	31		tt	gg	gg	tt			
77	-	-	+	-	112	5		tt	gg	gg	tt			
78	+	-	+	-	44	3		tt	gg	gg	tt			
79	+	+	+	-	30	0		tt	gg	gg	tt			
82								tt	gg	gg	tt			
83								tt	gg	gg	tt	202	63	109
91								tt	gg	gg	tt			
93	-	+	+	-	208	43		tt	gg	gg	tt			
94	+	-	+	-	516	153		tt	gg	gg	tt			
105								tt	gg	gg	tt			
106								tt	gg	gg	tt			
107								tt	gg	gg	tt			
13	+	-	+	+	14652	18393		cc	cg	tg	gt	251	76	164
14	-	+	+	+	6285	14271		cc	cg	tg	gt	168	55	100
67					6411	4099	6083	cc	cc	tt	gg			
68					6691	6133	2824	cc	cc	tt	gg			
69					6134	8567	4071	cc	cg	tt	gg			
71					4077	6620	7872	cc	cc	tt	gg			
102					7160	4087	922	cc	cc	tt	gg			
61	+	-	+	+	4602	1803		ct	cg	tg	gt			
62	+	-	+	+	3417	1284		ct	cg	tg	gt			
63	-	+	+	+	4191	651		ct	cg	tg	gt			
64	-	+	+	+	3432	1465		ct	cg	tg	gt			
65	+	+	+	+	3013	376	2922	ct	cg	tg	gt			
66					4077	1111	5568	ct	cg	tg	gt			
70					5517	1722	8425	ct	cg	tg	gt			
103					6441	3769	780	ct	cg	tg	gt			
104					7177	4491	843	ct	cg	tg	gt			

The genotypes are colour-coded as follows:

- $P^1 P^1$
- $P^1 P^2$
- $P^2 P^2$
- $p\,p$ (null)
- unassigned
- $P^{1NOR} P^1$
- NOR-negative family of NOR-positive individuals
- $P^{1NOR} P^2$
