# Supplementary material for: Single nucleotide polymorphisms in A4GALT spur extra products of the human Gb3/CD77 synthase and underlie the P1PK blood group system
Source: PLoS One. 2018 Apr 30;13(4):e0196627. doi: 10.1371/journal.pone.0196627 (PMC5927444; doi:10.1371/journal.pone.0196627)
Supplement: S2 Table — (PDF) [file pone.0196627.s004.pdf]

Table S2. Sequences of primers used for genotyping.

Name of primer	Sequence (5’→3’)
rs8138197sense	TGAATTAACCGAAAGAAGTAGG
rs8138197anti	CATAGCAAAATGCAAGCA
rsLaisense	GCATTCCTCATCGCAGAC
rsLaianti	ATAAATGCAGCCAAGTCTC
A4GTsense	GGAGAGCCCAAGGAGAAAG
A4GTanti	CAAGTACATTTTCATGGCCTC
