# Supplementary material for: Single nucleotide polymorphisms in A4GALT spur extra products of the human Gb3/CD77 synthase and underlie the P1PK blood group system
Source: PLoS One. 2018 Apr 30;13(4):e0196627. doi: 10.1371/journal.pone.0196627 (PMC5927444; doi:10.1371/journal.pone.0196627)
Supplement: S3 Table — (PDF) [file pone.0196627.s005.pdf]

Table S3. PCR conditions used for amplification of *A4GALT* fragments encompassing the studied SNPs.

Reaction step	Reaction parameters		
	**Temperature (°C)**	**Time (s)**	**Number of cycles**
Initial denaturation	94	180	1
Denaturation	94	30	29
Annealing	58	30	
Extension	72	150	
Final extension	72	600	1
