# Supplementary material for: Single nucleotide polymorphisms in A4GALT spur extra products of the human Gb3/CD77 synthase and underlie the P1PK blood group system
Source: PLoS One. 2018 Apr 30;13(4):e0196627. doi: 10.1371/journal.pone.0196627 (PMC5927444; doi:10.1371/journal.pone.0196627)
Supplement: S4 Table — (PDF) [file pone.0196627.s006.pdf]

Table S4. Real-time PCR conditions used for *A4GALT* gene expression assays.

**Real-time PCR System**	**Reaction format**	**Reaction volume**	**Thermal cycling conditions**			
			Parameter	Initial denaturation	PCR (42 cycles)	
					Denaturation	Annealing/ Extension
7500 Fast	96-well plate	20 µl	Temperature (°C)	95	95	60
			Time (s)	600	15	60
